# Case Report: Low-grade endometrial stromal sarcoma with intravenous and intracardiac extension: a case after misdiagnosis of endometrial stromal nodule as submucosal fibroid

**DOI:** 10.3389/fonc.2023.1205783

**Published:** 2023-10-16

**Authors:** Xiao-Shan Chai, Guang-Shi Tao, Hui Ding, Peng Zhou, Xi-Long Mei, Xiao-Xue Li

**Affiliations:** ^1^ Department of Obstetrics and Gynecology, The Second Xiangya Hospital of Central South University, Changsha, Hunan, China; ^2^ Department of Pathology, The Second Xiangya Hospital of Central South University, Changsha, Hunan, China; ^3^ Department of Radiology, The Second Xiangya Hospital of Central South University, Changsha, Hunan, China

**Keywords:** LG-ESS, intravenous and intracardiac extension, CD10, ESN, uterine fibroids, case report

## Abstract

We present herein a rare case of large vascular and cardiac metastases of low-grade endometrial stromal sarcoma (LG-ESS) in a female patient, which occurred after misdiagnosis of endometrial stromal nodule (ESN) as submucosal leiomyoma 7 years ago. Preoperative three-dimensional CT reconstruction was used to assess the extent of the lesion. The patient underwent radical resection: thrombectomy and total hysterectomy with bilateral salpingo-oophorectomy without establishing the cardiopulmonary bypass. Intraoperative transesophageal ultrasound (TEE) was used to monitor whether the intracardiac mass was removed completely. To date, this patient is alive without any evidence of recurrence 3 years after surgery. The differential diagnosis of ESN and LG-ESS is often difficult. A clear distinction can only be reliably made after histological analysis of the tumor’s entire interface with the neighboring myometrium. This case highlights that follow-ups of patients with ESN are important. Regular follow-up can detect metastasis and recurrence of misdiagnosed LG-ESS as early as possible. Distant metastasis of LG-ESS is rare, especially involving large vessels or the heart. The treatment should largely rely on multidisciplinary cooperation. Although the surgery is traumatic, the perioperative mortality rate is low, and patients can avoid death from congestive heart failure or sudden death.

## 1 Introduction

In the differential diagnosis of lesions in the inferior vena cava and within the heart, when thrombosis is excluded, lesions caused by the invasion of malignant tumors should be considered. Common tumors that may transfer to the inferior vena cava and the heart include renal cancer, liver cancer, uterine leiomyomatosis, and nephroblastoma (1). We here present a low-grade endometrial stromal sarcoma (LG-ESS) case with intravenous and intracardiac metastasis. In the current World Health Organization (WHO) classification (published in 2014), endometrial stromal tumors were classified into three types according to cell morphology and mitosis: benign endometrial stromal nodules (ESN), endometrial stromal sarcoma (ESS), and undifferentiated uterine sarcoma (UUS) (2). ESS was further classified into high grade (HG-ESS) and low grade. ESS is a very rare tumor entity, representing only around 0.2% of all uterine malignancies, but it makes up approximately 10% of uterine sarcomas (3). Preoperative diagnosis of LG-ESS is difficult because it is often confused with uterine fibroids (4). ESS usually spreads through the lymph nodes and venous system, but it seldom spreads to large vessels or the heart region. Cardiac metastasis is extremely rare in ESS, and its treatment is very complex (5, 6). Patients with heart involvement are often recurrent cases who have received surgery for endometrial stromal sarcoma (6–10). We here present a “primary” LG-ESS case with intravenous and intracardiac metastasis. However, due to the patient’s history of submucosal myoma resection 7 years ago, the pathological diagnosis was revised to ESN after the reconsultation of the pathological section from the last operation. LG-ESS cannot be completely ruled out due to the lack of sufficient pathological specimens. There is a high possibility that this case is a relapse of LG-ESS.

## 2 Case presentation

**Figure 1:**
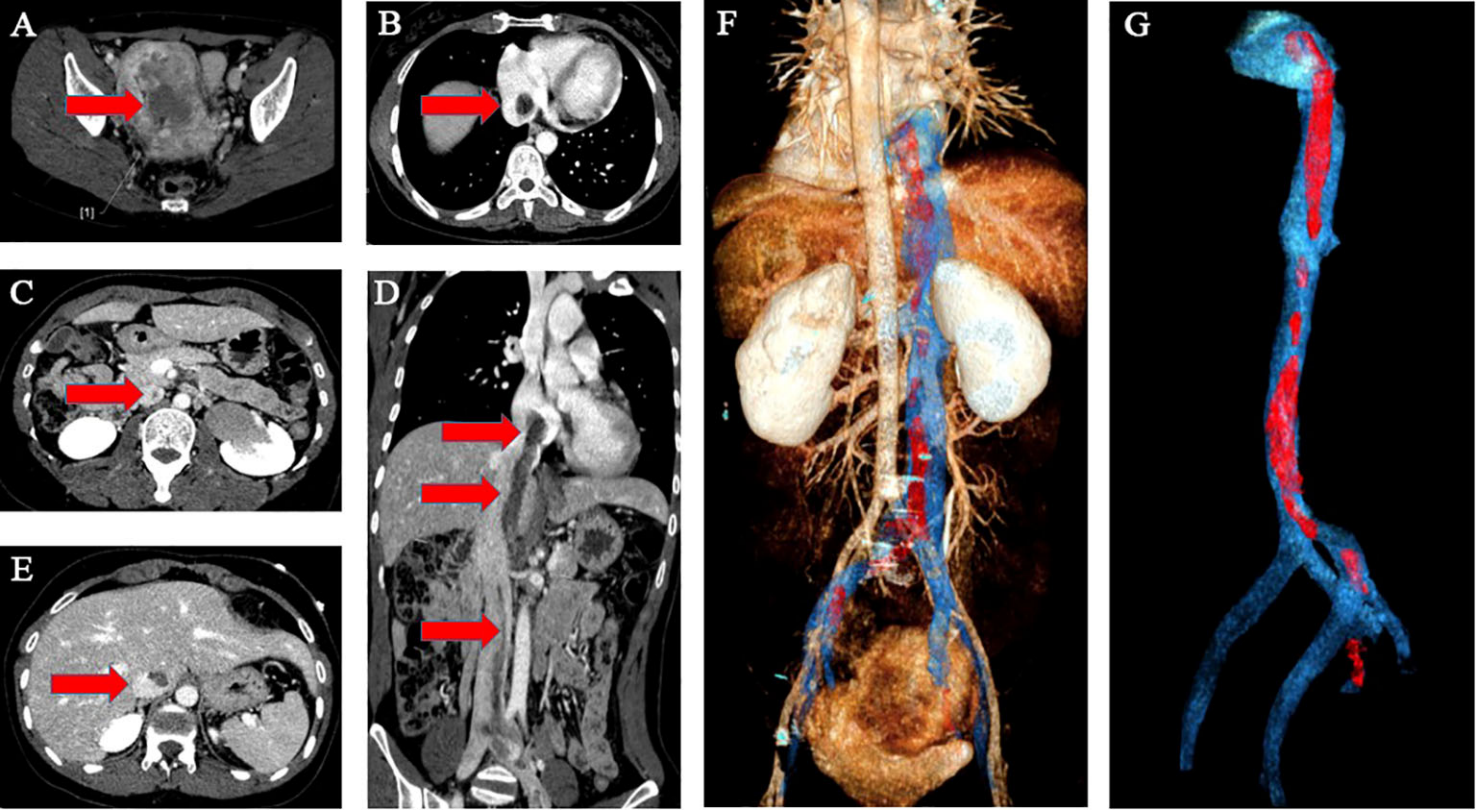
Imaging of the tumor. The superior and inferior vena cava CTA showed an enlarged uterine as well as low-density image in the venous system and the right atrium **(A–E)**. The red arrow indicates the mass. The three-dimensional computed tomography reconstruction of the whole path of the mass originating from the uterine and invaded into the venous system and eventually to the right atrium **(F, G)**. **(F)** Back view **(G)**. Endovascular imaging of the tumor. Red represents the mass; blue represents the blood vessel.

**Figure 2:**
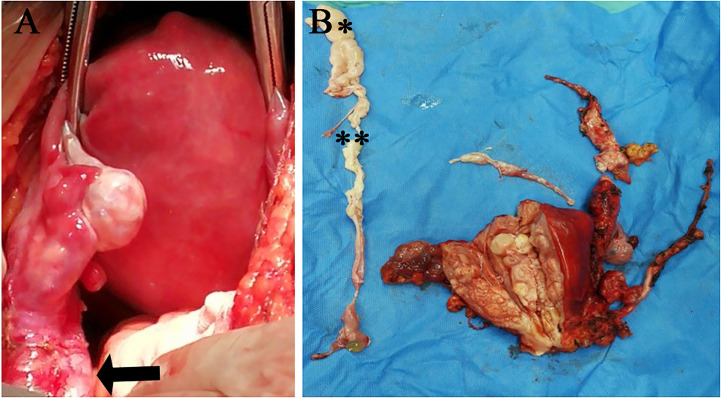
Imaging of the operation **(A)** and gross specimen **(B)**.The black arrow indicates the ovarian blood vessel dilated by the tumor. (^**^intravascular metastatic lesion; ^*^intracardiac metastatic lesion).

**Figure 3:**
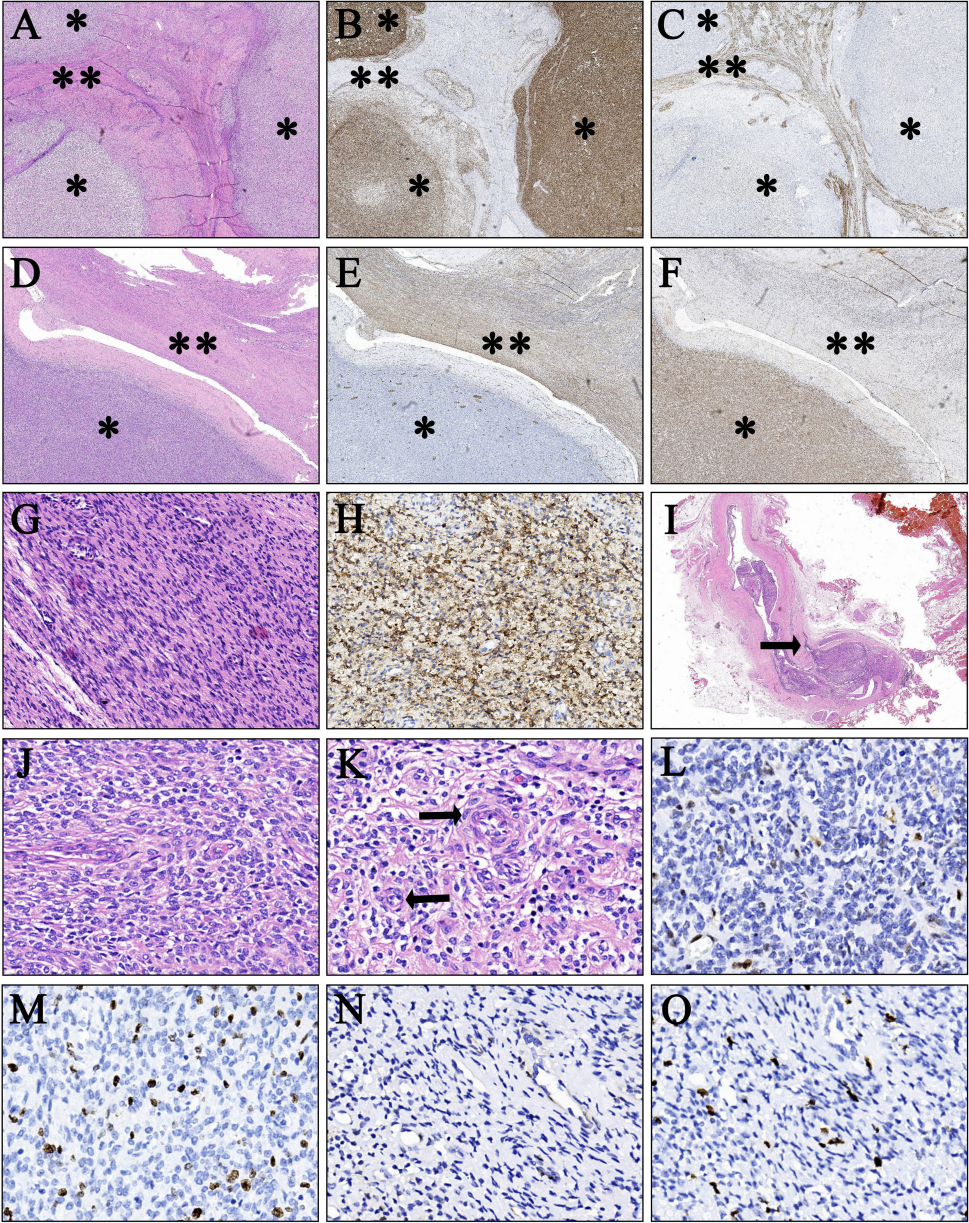
Pathological findings of the primary uterine foci and intravascular metastatic lesion. **(A–C)** Immunohistochemical findings of the primary uterine foci a (H&E, orig., ×15). Tumor cells are positive for CD10 but negative for Desmin. Smooth muscle tissues are positive for Desmin. (**B**, CD10; **C**, Desmin; ^**^smooth muscle component; ^*^tumor cell component). **(D–F)** Immunohistochemical findings of the primary uterine foci b (H&E, orig. ×20). Tumor cells are positive for ER but negative for SMA. Smooth muscle tissues are positive for SMA. (**E**, SMA; **F**, ER; ^**^smooth muscle component; ^*^tumor cell component). **(G, H)** Pathological findings of the intracardiac metastatic lesion (H&E, orig. ×200). Tumor cells are positive for CD10 (**H**, CD10). **(I)** Intravascular metastatic tumor tissue (H&E, orig. ×15). The black arrow indicates that the tumor tissue partially infiltrated the vein. **(J, K)** Cell morphology (H&E, orig. ×400). Photomicrograph shows a small and uniform population of round to oval cells with little–moderate eosinophilic cytoplasm and nuclear mitotic activity 0–1/400 HPF. The black arrow indicates the spiral artery. [**J**, local amplification of primary lesion a **(A)**; **K**, local amplification of primary lesion b **(D)**]. **(L)** Cyclin D1 (10%) staining in the primary uterine foci (H&E, orig. ×400). **(M)** Ki-67 (40%) staining in the primary uterine foci (H&E, orig. ×400). **(N)** Cyclin D1 (<1%) staining in metastatic lesions (H&E, orig. ×400). **(O)** Ki-67(4%) staining in metastatic lesions (H&E, orig. ×400).

**Figure 4:**
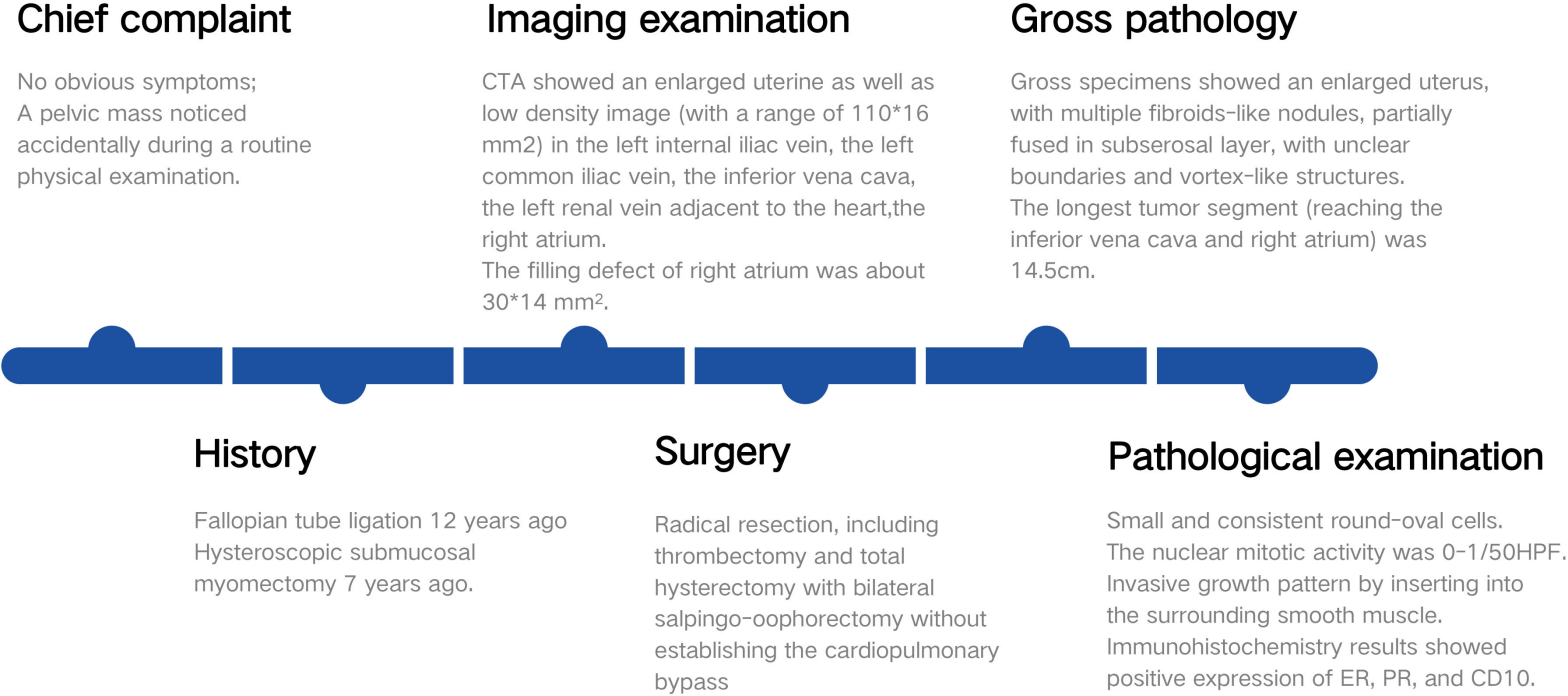
The therapeutic course of our patient.

A 38-year-old woman (gravida 3, para 2) was admitted to our hospital due to a pelvic mass noticed accidentally during a routine physical examination. The patient had no obvious symptoms, and the pelvic mass was found incidentally on physical examination. Further abdominal and pelvic ultrasound, as well as the cardiac echocardiography, showed inferior vena cava and right atrium lumps. Segmental curettage was performed to rule out endometrial lesions. A needle biopsy of the pelvic mass was performed, and the pathologic tissue showed smooth muscle-like morphology. Immunohistochemistry results showed positive expression of Ki-67 (2%+), Vim, S100, SMA, Desmin, and HHF35, and the biopsy sample had negative expression of CD117, ALK, and Dog-1. The past medical history included fallopian tube ligation 12 years ago and hysteroscopic submucosal myomectomy 7 years ago. When reassessing the pathological sections from the last operation 7 years ago, the diagnosis was revised to ESN; LG-ESS cannot be completely ruled out. She reported no family history of cancer. The patient’s temperature was 36.4°C, heart rate was 90 bpm, respiratory rate was 20 breaths per minute, blood pressure was 111/91 mmHg, and oxygen saturation in room air was 99%. The pelvic palpation revealed a 3-month pregnant-sized uterus without abnormalities in other reproductive organs. The blood and urine analyses were normal. Electrocardiogram, chest X-ray, and arterial blood gas were also normal. Prothrombin and partial thromboplastin times were normal, and D-dimers were increased at 1.72 μg/mL. The increase of D-dimer is considered a hypercoagulable state of the body caused by the tumor, as thrombotic disease has been ruled out in this patient. Blood testing showed hyperlipemia, with cholesterol at 6.28 mmol/L and triglycerides at 1.79 mmol/L. Blood examination also showed slightly elevated levels of trioxypurine (366 U/L) and cancer antigen (CA)125 (102.23 U/mL), and the CA19–9 level was normal. The cervical cytology was normal. The transvaginal ultrasonography revealed an enlarged uterus as large as 3 months of pregnancy. The superior and inferior vena cava CT angiography (CTA) showed an enlarged uterine as well as low-density image (with a range of 110 * 16 mm^2^) in the left internal iliac vein, the left common iliac vein, the inferior vena cava, the left renal vein adjacent to the heart, and the right atrium. The filling defect of the right atrium was about 30 * 14 mm^2^. The tumor extended along the veins, and the venous system shows low-density shadows (**Figures 1A-E**). The three-dimensional computed tomography reconstruction showed that the mass might have originated from the uterine and invaded the reproductive vein, subsequently extending along the inferior vena cava to the right atrium (**Figures 1F, G**). FDG-positron emission tomography/computer tomography (PET-CT) demonstrated a maximum standardized uptake value of 14.5 confined to the uterine masses in contrast to an uptake value of 8.5 in the intravascular and intracardiac metastatic masses. With full disclosure and informed consent, the patient underwent radical resection, including thrombectomy and total hysterectomy with bilateral salpingo-oophorectomy without establishing cardiopulmonary bypass. Intraoperative transesophageal echocardiography (TEE) showed that the atrial tumor was completely removed, and successively, the tumors in the vena cava and the left common iliac vein, internal iliac vein, and left renal vein were removed. Gross specimens showed an enlarged uterus with multiple fibroid-like nodules, partially fused in the subserosal layer, with unclear boundaries and vortex-like structures. The longest tumor segment (reaching the inferior vena cava and right atrium) was 14.5 cm (**Figures 2A, B**). Postoperative histological morphology showed a group of small and consistent round-oval cells. Unlike HG-ESS, the nuclear mitotic activity of tumor cells was 0–1/400 HPF. Moreover, little-medium eosinophilic cytoplasm and small spiral artery differentiation were recognized. Different from ESN, the tumor tissue showed an invasive growth pattern by inserting into the surrounding smooth muscle (**Figures 3G-K**). Immunohistochemistry results showed positive expression of ER and CD10 but negative for Desmin and SMA of tumor cells(**Figures 3A-F**). The primary uterine foci showed 10%+ cyclin D1 and 40%+ Ki-67 expression (**Figures 3L, M**), whereas the metastatic lesions (intracardiac and intravascular lesions) showed negative cyclin D1 and 4%+ Ki-67 expression (**Figures 3N, O**). Overall, histomorphology and immunohistochemistry analysis confirmed the LG-ESS diagnosis but not HG-ESS nor uterine fibroids. The therapeutic course of our patient is shown in **Figures 4**. The patient had an uneventful postoperative clinical course and was discharged 12 days after surgery. Treatment with the aromatase inhibitor letrozole (2.5 mg/day) was continued. Three months after the operation, the patient reported full recovery and was able to engage in normal work and activities. Moreover, an MRI examination showed no recognizable signs of recurrence 3 years after surgery.

## 3 Discussion

As a very rare malignant gynecological neoplasm, the annual incidence of ESS is 0.19 per 100,000 women (7). LG-ESS has an indolent history with a recurrence rate of 10%–20%, and it generally reoccurs many years after diagnosis and the initial surgery (5, 11). According to the literature, the most frequent metastasis sites of the LG-ESS are the vagina, pelvis, and peritoneal cavity, while cardiac metastases are very rare because ESS commonly spreads through the lymph nodes and venous system (5). To our knowledge, 18 LG-ESS patients with heart involvement have been reported to date (5–10, 12–23). The age of the 18 patients with heart involvement ranged from 24 to 71 years old (average age: 46 years), 66.7% had a history of surgery for LG-ESS, and only 33.3% were diagnosed for the first time. Our case differs from other cases of cardiac metastasis in that although this is the first time the patient has been diagnosed, the patient had a history of submucosal myomectomy 7 years ago, when reassessing the pathological sections from the last operation 7 years ago, ESN should be considered. Due to the limited amount of specimens, the diagnosis of LG-ESS cannot be ruled out. It is likely that the patient already had LG-ESS 7 years ago, and the tumor relapsed this time.

ESN is the least common type of endometrial stromal tumor (24), characterized and defined as benign and noninvasive. Hysterectomy is required to distinguish it from LG-ESS; therefore, hysterectomy is the preferred treatment for patients with ESN (25). Our patient was misdiagnosed as submucosal myoma 7 years ago, which was actually ESN. However, the prognosises of several patients with ESN were reviewed, and no obvious signs of recurrence were found (26). Since total hysterectomy was not performed in this patient, the lesion and surrounding muscle layer infiltration could not be determined, so ESN and LG-ESS could not be distinguished. Su et al. have reported cases of ESN with infiltration potential (27). It is possible that our patient was an LG-ESS patient 7 years ago or had ESN with infiltration potential; the metastases of the heart and large vessels were due to recurrence of LG-ESS or ESN with infiltration potential.

Preoperative diagnosis of LG-ESS is difficult; it can be easily confused with uterine fibroids or ESN because of its limited, specific clinical manifestations (5). Imagological procedures such as ultrasound, computed tomography, and magnetic resonance imaging are also not able to display any specific characteristics of LG-ESS (28). Some scholars point out that MRI or PET-CT is not useful in the differentiation of ESN from LG-ESS (29). It has been proposed recently that MRI showed a specific nodule-in-nodule appearance in LG-ESS and suggests that preoperative diagnostic imaging with MRI may be useful (30). However, the pathological diagnosis remains the gold standard.

There have been numerous reports of uterine fibroids spreading intravascularly. Although MRI is a useful tool for distinguishing between benign leiomyoma and sarcoma, degenerated benign leiomyomas may have findings similar to those of sarcomas. In our case, a needle biopsy of the pelvic mass was performed, and the pathologic tissue showed smooth muscle-like morphology. The diagnosis of intravascular leiomyomatosis cannot be ruled out. It is crucial to diagnose by surgical resection. Although the tumor cells in our case showed smooth muscle differentiation, the immunohistochemistry supported LG-ESS rather than leiomyoma.

There are two implications from this case: Firstly, pathologists should pay more attention to distinguishing the diagnosis of uterine fibroid from ESN. Secondly, for patients diagnosed with ESN, hysterectomy should be recommended. If the uterus is not removed, close follow-up should be conducted to avoid distant metastasis of the tumor.

The clinical symptoms of LG-ESS involving the heart and great vessels vary from asymptomatic to obvious dyspnea and right heart failure. CT and MRI examinations are powerful tools for early detection of cardiac and vascular involvement. However, there are also cases in which CT or MRI were unable to detect the free−floating intravascular tumor but were detected accidentally by TEE (12, 22), suggesting that for patients with possible major vascular metastasis, TEE should be stressed and applied as early as possible. Several case reports pointed out that TEE can also guide the doctor to determine whether the cardiac mass is removed completely during the surgery (6). In the present report, three-dimensional CT reconstruction imaging was used to simulate the tumor route and scope, which helped evaluate the range of the lesion, and also intraoperative TEE was used to guide the complete resection of intraoperative cardiac tumor.

The primary treatment for LG-ESS is surgery with total hysterectomy and bilateral salpingo-oophorectomy (31). It has been shown that LG-ESS is hormone-dependent. It is not clear whether the ovaries can be preserved in young, premenopausal women (32). There is no evidence that cytoreduction and lymphadenectomy are beneficial for long-term survival (33). Among the 19 patients with cardiac involvement (including our case), radical resection were performed in 15 cases, and the perioperative mortality rate was zero. All patients recovered uneventfully after the operation. Except for one case, all addressable patients showed no signs of recurrence by the end of the follow-up. The prognosis is good even if the operation is extremely invasive, such as open heart surgery under extracorporeal circulation. The key to treatment is a thorough surgical removal of the lesions, especially tumors in the heart. On the contrary, the prognosis of incomplete surgery is poor. One patient died after unsatisfactory tumor reduction surgery (subtotal resection of cardiac thrombus) (13), three patients did not receive surgery, one of whom died of heart failure 14 years later (16), and two of whom lived with tumors at the time of publication of the article (6 months follow-up) (5, 10). Although the long-term effect of surgery is still unclear, it is definite that surgery can prevent patients from developing severe complications, such as heart failure and pulmonary embolism, and even sudden death.

There are no valid data supporting the survival benefits of adjuvant chemotherapy in LG-ESS (31). Postoperative radiotherapy only improves locoregional control; therefore, medium- and long-term side effects of pelvic irradiation and their efficacy against recurrences need to be carefully weighed (34). The expression of steroid receptors and aromatases in LG-ESS suggests that adjuvant therapy with gestagens, GnRH analogs, or aromatase inhibitors should be effective (31).

In the existing reports, PET-CT is mostly used in the evaluation of the effect of postoperative chemotherapy and anti-angiogenic therapy for LG-ESS with multiple metastases (35, 36). In our case, an interesting finding is that immunohistochemistry findings of cyclin D1 and Ki-67 are different between the primary uterine foci and the metastatic lesions (the intracardiac and the intravascular component). The preoperative PET-CT SUVmax of the primary lesion was about 14.5, and that of the metastatic focus was about 8.5. The immunohistochemical results of CyclinD1 and Ki-67 were in accordance with the SUVmax of PET-CT. This result is consistent with the study of Fujiishi et al. (36). The two cell cycle proteins CyclinD1 and KI-67 of the same tumor showed different levels of expression in different lesions, and this may explain the different responses within different regions under chemotherapeutic agents targeting cell division. Some masses may shrink by nearly 80%, while others may have no significant change (17). Based on the above conclusions, we believe that PET-CT as well as the immunohistochemical results of CyclinD1 and Ki-67 can be informative to guide subsequent treatment such as chemotherapy and antiangiogenic therapy. However, more evidence needs to be further accumulated.

Still, this study has some limitations. We have limited information about the tumor cell features so far, especially the gene mutations that are relatively common in ESS such as JAZF1/SUZ12, JAZF1-PHF1, and EPC1-PHF1 fusions (37), micro-satellite stability characteristics, sensibility toward chemotherapeutic and target drugs, and capability of angiogenesis. Our further work will conduct *in vitro* experiments and explore more aspects of its features. Moreover, the follow-up period is not long enough, and the 5-year survival outcome is unknown.

## 4 Conclusion

Cardiac metastasis of LG-ESS is rare, and the treatment should focus on reducing the tumor load, especially by removing lesions that have spread to the heart and major vessels. This frequently calls for multidisciplinary cooperation. The resection of the lesion is of great importance to the prognosis of the patient. In particular, gynecologic oncologists and pathologists should carefully distinguish between “uterine leiomyoma”, “ESN”, and “LG-ESS” and pay attention to the follow-up of high-risk patients.

## Data availability statement

The original contributions presented in the study are included in the article/supplementary material. Further inquiries can be directed to the corresponding author.

## Ethics statement

The studies involving humans were approved by The Human Ethics Committee of The Second Xiangya Hospital of Central South University (No.2019(218)). The studies were conducted in accordance with the local legislation and institutional requirements. The participants provided their written informed consent to participate in this study. Written informed consent was obtained from the individual(s) for the publication of any potentially identifiable images or data included in this article.

## Author contributions

X-SC designed the article and wrote the manuscript. G-ST, HD, and X-SC performed the surgery. PZ conducted the histopathological and immunohistochemical analysis. X-LM conducted the imageological analysis. X-XL analyzed the patient data and followed up on patient information. G-ST was responsible for the revision of the manuscript for important intellectual content. All authors contributed to the article and approved the submitted version.
